# Pattern Recognition Methods and Features Selection for Speech Emotion Recognition System

**DOI:** 10.1155/2015/573068

**Published:** 2015-08-04

**Authors:** Pavol Partila, Miroslav Voznak, Jaromir Tovarek

**Affiliations:** Department of Telecommunications, Faculty of Electrical Engineering and Computer Science, VSB-Technical University of Ostrava, 17 Listopadu 15, 70833 Ostrava, Czech Republic

## Abstract

The impact of the classification method and features selection for the speech emotion recognition accuracy is discussed in this paper. Selecting the correct parameters in combination with the classifier is an important part of reducing the complexity of system computing. This step is necessary especially for systems that will be deployed in real-time applications. The reason for the development and improvement of speech emotion recognition systems is wide usability in nowadays automatic voice controlled systems. Berlin database of emotional recordings was used in this experiment. Classification accuracy of artificial neural networks, *k*-nearest neighbours, and Gaussian mixture model is measured considering the selection of prosodic, spectral, and voice quality features. The purpose was to find an optimal combination of methods and group of features for stress detection in human speech. The research contribution lies in the design of the speech emotion recognition system due to its accuracy and efficiency.

## 1. Introduction

The development of applications and services is trying to deploy natural interaction between man and computer. Specifying commands by voice and movements is very popular nowadays. The majority of information is extracted from human speech with rather good accuracy. Human speech also includes secondary information, which holds properties of the speaker. Age, gender, emotional state, speech error, and other features are contained in human speech. The mentioned source characteristics are highly valuable, because speech features can be simulated only by person with good acting skills. As the title suggests, this paper describes a system for classifying emotional state of human speech. Emotion is one of the characteristics of human which describes his mental condition affecting physiological changes in the human body. These changes are also reflected in the human speech. Information about the emotional state is requested in many fields. Statistical evaluation of customer satisfaction and his interest in the products is evaluated by affected emotional state. This information is a direct response to any stimulus. Call center agents can be evaluated with regard to their work and access to the customer. There is a chance to train new agents and teach them to correct the procedure of communication with the customer. Human body influenced by stronger emotions is getting stressed. Sectors such as police, firemen, and especially military generate the greatest emotional pressure on employees. Dispatching orders can be directly influenced by information from speech emotion recognition system. Speech signal can serve as an authorization key in access systems. Speech is affected by physiological changes caused by changing emotions. An authorized user can be denied because authorization unit recognizes the stress speech as a wrong key. These are just the first examples of utilizations for speech emotion recognition systems. It is obvious that the system will have great application in human-machine interaction. Therefore it is appropriate to identify a classification ability of different classifiers for different emotional states. One of the related works, but a more extensive research summary from Mr. El Ayadi et al., is published in the article “survey on speech emotion recognition: features, classification schemes, and databases,” which is mentioned in [1–4].

## 2. Speech Emotion Recognition System

**Figure 1:**
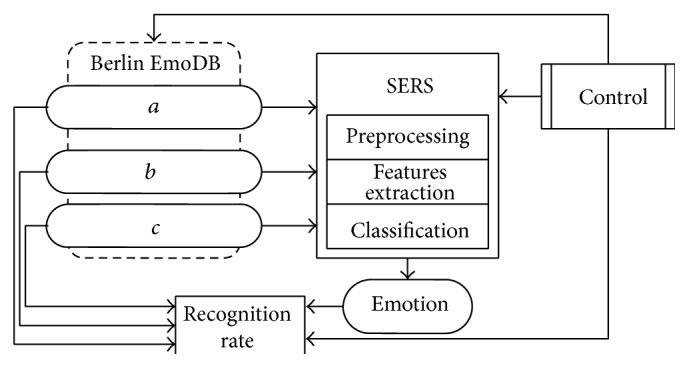
Block diagram of speech emotion recognition system (SERS). The system consists of a database that is used for training and testing and other blocks that describe functions of the algorithm. Different views on the issue are represented by blocks. (*a*) Stress versus neutral state classification, (*b*) each kind of emotion, and (*c*) other approaches. Scenario option is represented by control block.

System design consists of several blocks, which it distributed to major functions. Input values are represented by audio signals from created database and used for training and testing. Block diagram of the system is shown in Figure 1.

The quality of the input data, the audio signal in this case, has a direct impact on the classification accuracy. For this reason, the Berlin database containing over 500 recordings of actors consisting of men and women is used. The database contains 10 sentences in the seven emotional states. This corpus of recordings is considered as a high-quality database, because it was created by professional actors in the sound studio. Blocks *a*, *b*, and *c* represent point of view for emotion recognition. The system can be designed and used for detecting the stress of the speaker (option *a*), for recognition of all emotional states, as in the case of the Berlin database in which they are seven (option *b*). Other approaches to the problem are represented by block *c*. As mentioned, the speech signal has to be modified by routine preprocessing operations such as removing the DC component, preemphasis, and segmentation stochastic signal into quasiperiodic frames.

Speech recognition system is context independent, that is, taking into account only signal parameters, not content information. These parameters are the training and testing vectors for classifiers [5–7].

The calculation parameters are represented by the features extraction block that extracts the following:

- 39 Mel-frequency cepstral coefficients (MFCC) and dynamic parameters (first and second derivative of MFCC),
- 12 linear prediction coefficients (LPC),
- 12 linear spectral pairs (LSP),
- 8 prosodic features (RMS energy, log-energy, zero crossing rate (ZCR), mean crossing rate (MCR), position of maximum, maximum, minimum, and harmonic-to-noise ratio (HNR) [8].

## 3. Classifiers

Individual research shows that it cannot be said which classifier for emotion recognition is the best. Each classifier or their combination achieved some results accuracy, which depends on several factors. The success of classifier is directly dependent on the data. This is derived from the fact that the accuracy varies with the data character such as the quantity, density distribution of each class (emotions), and the language too. One classifier has different results with acted database, where the density of each emotion is equitable and different with real data from call center where normal (calm) emotion state occupies 85 to 95 percent of all data. Appropriate choice of parameters has a considerable effect on the accuracy of these classifiers. The following subsections describe the used classification methods.

### 3.1. Artificial Neural Network

Our emotional state classification problem with high number of parameters can be considered as a pattern-recognition problem. In this case, two-layer feedforward network can be used. A two-layer feedforward network, with sigmoid hidden and output neurons, can classify vectors arbitrarily well, given enough neurons in its hidden layer. The network is trained with scaled conjugate gradient (SCG) backpropagation.

**Figure 2:**
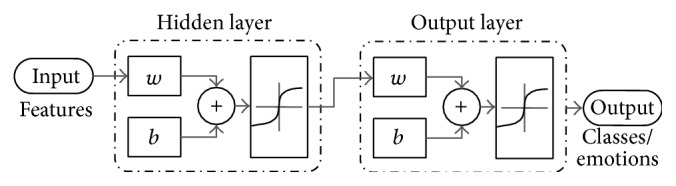
Artificial neural network architecture with hidden layers and output classes.

We shall denote the input values to the network by *x*_*i*_ where *i* = 1,…, *d*. The first layer of network forms *M* linear combinations of these inputs to give a set of intermediate activation variables *a*_*j*_^(1)^:

(1) $$\begin{array}{c} a_{j}^{\left(1\right)}=\overset{d}{\underset{i=1}{\sum }}w_{ij}^{\left(1\right)}x_{i}+b_{j}^{\left(1\right)},\;j=1,\ldots ,M, \end{array}$$

with one variable *a*_*j*_^(1)^ associated with each hidden unit. Here *w*_*ij*_^(1)^ represents the elements of first-layer weight matrix and *b*_*j*_^(1)^ is the* bias* parameters associated with the hidden units. Demonstration of such a network with speech features as an input, 5 hidden layers, and two output classes is shown in Figure 2.

SCG training implements mean squared error *E*(*w*) associated with gradient ∇*E* and avoids the line search per learning iteration by using Levenberg-Marquardt approach in order to scale the step size. A weight in the network will be expressed in vector notation:

(2) $$\begin{array}{c} w=\left(\ldots ,w_{ij}^{\left(1\right)},w_{i+1j}^{\left(1\right)},\ldots ,w_{N_{1}j}^{\left(1\right)}\theta_{j}^{\left(l+1\right)},w_{ij+1}^{\left(1\right)},w_{i+1j+1}^{\left(1\right)},\ldots \right). \end{array}$$

The vector-delta *E* points in the direction in which *E*(*w*) will decrease at the fastest possible rate. Weight update equation is shown below, where *c* is suitable constant:

(3) $$\begin{array}{c} w\left(k+1\right)=w\left(k\right)-c\nabla E. \end{array}$$

The gradient descent method for optimization is very simple and general. Only local information, for estimating a gradient, is needed for finding the minimum of the error function [9, 10].

### 3.2. **k**-Nearest Neighbour

The *k*-NN is a classification method on the principle of analogies learning. Samples from the training set are *n* numeric attributes, and each sample represents a point in *N*-dimensional space. This space of training samples is scanned by the classifier due to determining the shortest distance between training and unknown samples. Euclidean and other distances can be computed. In other words, an object is classified by a majority vote of its neighbours, with the object being assigned to the class most common amongst its *k* nearest neighbours (*k* is a positive integer, typically small). If *k* = 1, then the object is simply assigned to the class of its nearest neighbour. The various distances between the vectors *x*_*i*_ and *y*_*i*_ are as follows:

(4) $$\begin{array}{c} d\left(X,Y\right)=\sqrt{\overset{n}{\underset{i=1}{\sum }}{\left(x_{i}-y_{i}\right)}^{2}}. \end{array}$$

The neighbourhood distance is calculated through Euclidean metric. Given an *m*-by-*n* data matrix *X*, it is treated as *m* (1-by-*n*) row vectors *x*_1_, *x*_2_,…, *x*_*m*_.

### 3.3. Gaussian Mixture Model

A Gaussian mixture model is a parametric probability density function represented as a weighted sum of Gaussian component densities. GMMs are commonly used as a parametric model of the probability distribution of continuing measurements or features in biometric system, such as vocal tract, in speaker recognition systems as well. Probability distribution of the parameter vectors derived from human speech can be described using GMM:

(5) $$\begin{array}{c} p\left(o\;|\;\lambda^{s}\right)=\overset{M^{s}}{\underset{i=1}{\sum }}‍w_{i}^{s}p_{i}^{s}\left(o\right), \end{array}$$

where *M* is the number of components for *s* class, *w*_*i*_, *i* = 1,…, *M* are weights of components complying the condition that sum of all weights is 1, and *p* means the probability density of the components represented by the mean value and covariance matrix *C*_*i*_. Gaussian model for class “*s*” is defined by (6)

(6) $$\begin{array}{c} \lambda^{s}=\left\{w_{i}^{s},\mu_{i}^{s},C_{i}^{s}\right\},\;i,\ldots ,M^{s}. \end{array}$$

The criterion of maximum likelihood depends on the probability density *p* and sequence parameters *O* = (*o*_1_, *o*_2_,…, *o*_*n*_), as seen bellow [11, 12]:

(7) $$\begin{array}{c} \lambda^{s}=\arg\max p\left(o\;|\;\lambda^{s}\right). \end{array}$$

## 4. Experiment

The aim of the experiment was to clarify the significance of chosen groups of features, as well as classification ability of selected classification methods for speech emotion recognition system. Samples of examination were formed from recordings of human speech with various emotional characters. The following settings and features were used in the experiment:

(i) input samples—Berlin database of emotional utterances:
  (a) 10 different sentences recorded by 10 different actors (both genders),
  (b) over 530 samples consisting of 7 emotions:* anger, boredom, disgust, fear, happiness, sadness, neutral state*;
(ii) feature extraction—computing of input vectors (speech parameters):
  (a) 13 MFCC coefficients *c*_*m*_ = [*c*_*m*_(0),…, *c*_*m*_(12)], 13 dynamic Δ*c*_*m*_, and 13 acceleration coefficients of MFCC Δ^2^ *c*_*m*_,
  (b) 12 LPC coefficients,
  (c) 12 LSP coefficients,
  (d) 8 prosodic features;
(iii) emotion classification:
  (a) GMM—64 mixture components,
  (b) *k*-nearest neighbours (set up 5 neighbours);
  (c) artificial neural network—feedforward backpropagation.

## 5. Result

One of the points of view is recognizing the stressed-out person, which means recognizing deviations from the neutral state. This state is not defined in the Berlin database. Therefore, it was necessary to assemble a set of data, the so-called “stress cocktail” from defined emotional states.

The stress of a person can be assembled from emotional states, other than neutral state. Emotions, anger and fear, were used to compile the stress data set with 50/50 ratio that these emotional states are reflected most often when a person is exposed to stressful situations. Fear and anger were selected because of the major sound differences from neutral state. Confusion matrices for each set of features are shown in Tables 2, 3, 4, and 5. The meaning of cells describes Table 1. True positive (TP) represents correctly classified first class (neutral) and true negative (TN) correctly classified second class (stress).

**Figure 3:**
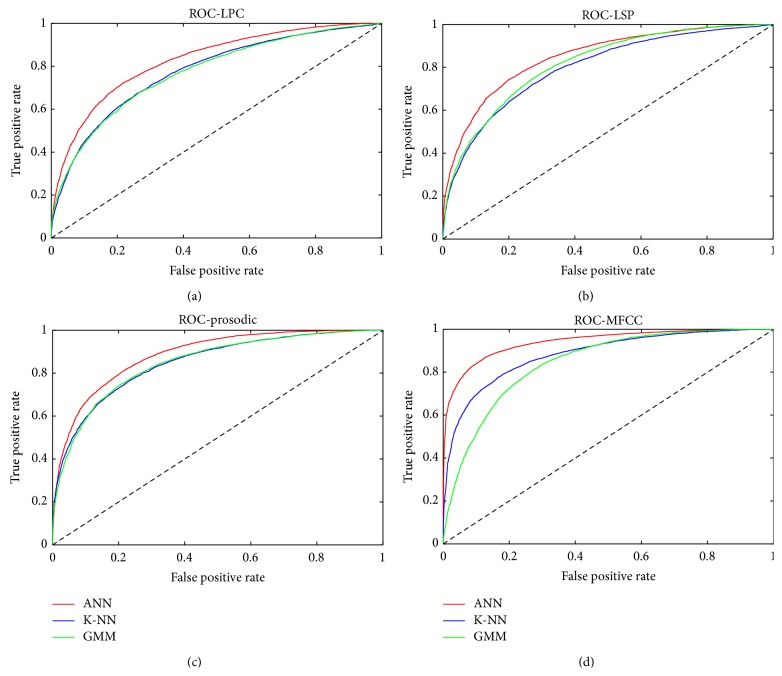
Receiver operating characteristic of GMM, *k*-NN, and ANN classifier in neutral state versus stress recognizing for LPC, LSP, prosodic, and MFCC features.

The GMM, *k*-NN, and the ANN were used to classify the stress versus neutral state. Results for all three classifiers are shown in Figure 3. The receiver operating characteristic (ROC) is applied for better system understanding. ROC curve is a tool for the evaluation and optimization of binary classification system (test), which shows the relationship between the sensitivity and specificity of the test or the detector for all possible threshold values [13].

The results in Tables 6, 7, and 8 describe classification accuracy for a particular type of classifier that has been trained by best-scored MFCC features of emotion pair. The classifier was trained by pair of emotions and values in the tables show tested ability of recognizing emotional state (left table header). All three classifiers showed the best recognition ability for the emotional state of anger. Emotional state of sadness was recognized with the evaluation very well. On the other hand, the worst-recognition ability of the system was the emotional state of fear (GMM and ANN) and disgust (ANN).

## 6. Conclusion

Neutral state versus stress scenario has been used for evaluating the accuracy of classification methods and features. The results show that the most precise method for recognizing speech of the human stress state is an artificial neural network, which achieved the best results for all sets of parameters (90% for MFCC). The most significant feature for emotion classification is MFCC. This fact demonstrates accuracies of all the classifiers and the ratio of the sensitivity and specificity of the ROC curve shown in Figure 3. One of the reasons is the individuality of MFCC coefficients, which are not mutually correlated.

This experiment shows that these classification methods can be used on the recognition of emotional state. At the same time, the question arises: what emotional states will characterize stress. The answer will probably depend on which system would be applied. Another fact is that we cannot determine the intensity of emotionally stimulated Berlin database. One of the main tasks will be to compare these results with the emotional recordings of the realistic environmental conditions.

## Figures and Tables

**Table 1:** Confusion matrix: description of cells.

Classifier			

Output classes	True positive	False positive	Positive predictive value
	False negative	True negative	Negative predictive value

	Sensitivity	Specificity	Precision
	Target classes		

**Table 2:** Performance results of classifiers for LPC features.

LPC	ANN			*k*-NN			GMM		
Neutral state	1718 5.1%	920 2.7%	65.1%	2233 6.6%	3241 9.6%	40.8%	5240 15.6%	6023 17.9%	46.5%

Stress	7542 22.5%	23410 69.7%	75.6%	7027 20.9%	21089 62.8%	75.0%	4020 12.0%	18307 54.5%	81.9%

	18.6%	96.2%	74.8%	24.1%	86.7%	69.4%	56.6%	75.2%	73.4%
	Neutral state	Stress		Neutral state	Stress		Neutral state	Stress	

**Table 3:** Performance results of classifiers for LSP features.

LSP	ANN			*k*-NN			GMM		
Neutral state	3466 13.4%	1814 7.0%	66.1%	3409 13.2%	3369 13.0%	50.3%	5778 17.2%	5452 16.2%	51.5%

Stress	5794 22.4%	14820 57.2%	71.7%	5851 22.6%	13265 51.2%	69.4%	3482 10.4%	18818 56.2%	84.4%

	37.4%	89.1%	70.6%	36.8%	79.7%	64.4%	62.4%	77.6%	73.4%
	Neutral state	Stress		Neutral state	Stress		Neutral state	Stress	

**Table 4:** Performance results of classifiers for prosodic features.

LSP	ANN			*k*-NN			GMM		
Neutral state	4470 13.3%	1699 5.1%	72.5%	3953 11.8%	2503 7.5%	61.2%	6483 19.3%	4774 14.2%	57.6%

Stress	4790 14.3%	22629 67.4%	82.5%	5307 15.8%	21825 65.0%	80.4%	2777 8.3%	19556 58.2%	87.6%

	48.3%	93.0%	80.7%	42.7%	89.7	76.7%	70.0%	80.4%	70.1%
	Neutral state	Stress		Neutral state	Stress		Neutral state	Stress	

**Table 5:** Performance results of classifiers for MFCC (dynamic and acceleration coefficients too) features.

MFCC	ANN			*k*-NN			GMM		
Neutral state	7445 22.1%	1901 5.7%	79.7%	4919 14.6%	2209 6.6%	69.0%	6305 18.8%	3507 10.4%	63.3%

Stress	1684 5.0%	22587 67.2%	93.1%	4210 12.5%	22279 66.3%	84.1%	2824 8.4%	20981 62.4%	88.1%

	81.6%	92.2%	89.3%	53.9%	91.0%	80.9%	69.1%	85.7%	81.2%
	Neutral state	Stress		Neutral state	Stress		Neutral state	Stress	

**Table 6:** Gaussian mixture model classification accuracy for different combinations of emotions [%].

Train 2/test	Train 1						
	Anger	Boredom	Disgust	Fear	Happiness	Sadness	Neutral state
Anger	—	91.7	85.7	83.4	70	96.4	90.8
Boredom	76.3	—	64.9	66.2	71.9	65.3	59
Disgust	59.1	64.3	—	62.5	56	78.5	64.7
Fear	52.4	72.6	59.7	—	47.2	81.8	70.4
Happiness	42.7	83.9	73.7	73.9	—	90.5	82.4
Sadness	87.1	67	75.5	73	85.5	—	75
Neutral state	78.6	53.3	69.1	63.9	74	65.7	—

**Table 7:** *K*-nearest neighbours classification accuracy for different combinations of emotions [%].

Train 2/test	Train 1						
	Anger	Boredom	Disgust	Fear	Happiness	Sadness	Neutral state
Anger	—	91.2	90.3	89.8	81.1	94.7	92.4
Boredom	92	—	70.4	67.7	75.9	61.6	64.6
Disgust	49	55	—	59.7	51.8	71.2	56.6
Fear	46.9	60.3	56.5	—	47.8	75.1	68.2
Happiness	31.9	78	72.7	73.1	—	86.1	78.7
Sadness	89.2	70.5	84.8	79.7	89.6	—	81.1
Neutral state	79.8	40.5	70.6	66	77.4	65.7	—

**Table 8:** Feed forward backpropagation neural network classification accuracy for different combinations of emotions [%].

Train 2/test	Train 1						
	Anger	Boredom	Disgust	Fear	Happiness	Sadness	Neutral state
Anger	—	95.8	93.7	92.4	87.9	98.1	96.7
Boredom	92	—	87.6	83.4	91.2	75.1	77.1
Disgust	79.8	79.1	—	68.2	77.7	86.4	77.6
Fear	69.6	70.6	73.7	—	68.2	82.2	76.2
Happiness	32.3	88.3	79.8	83.9	—	95	88.9
Sadness	97.9	81.5	92.6	95.3	97.5	—	85.2
Neutral state	93	49.9	86	85.1	88.2	52.4	—
